# Lower-limb muscle activation patterns during the taekwondo roundhouse kick in elite and youth athletes: a functional principal component analysis

**DOI:** 10.3389/fbioe.2026.1844590

**Published:** 2026-06-11

**Authors:** Jianbo Sun, Jingyuan Sun, Gang Sun, Liang Yin, Shazlin Shaharudin

**Affiliations:** 1 School of Health Sciences, Universiti Sains Malaysia, Kota Bharu, Malaysia; 2 International Education College, Harbin University of Commerce, Harbin, China; 3 Science of Learning in Education Centre, National Institute of Education, Nanyang Technological University, Singapore, Singapore

**Keywords:** lower-limb biomechanics, neuromuscular control, roundhouse kick, surface electromyography, taekwondo

## Abstract

**Objective:**

The taekwondo roundhouse kick is a fundamental and performance-critical technique that requires precise lower-limb muscle coordination. However, previous studies have mainly focused on discrete electromyographic variables, providing limited insight into waveform-level variations across the kicking cycle, particularly between athletes of different age and training levels. Therefore, this study aimed to investigate lower-limb surface electromyographic (sEMG) waveform differences during the roundhouse kick between elite and youth taekwondo athletes using functional principal component analysis (FPCA).

**Methods:**

Thirty-eight male taekwondo athletes were included (19 elite, 19 youth). Surface electromyography was recorded bilaterally from seven trunk and lower-limb muscles, including the rectus abdominis, gluteus maximus, rectus femoris, biceps femoris, semitendinosus, lateral gastrocnemius, and tibialis anterior, and three-dimensional kinematic data were collected synchronously during standardized roundhouse kicks. Time-normalized sEMG waveforms were analyzed using FPCA to identify dominant and secondary modes of variation. Group differences in principal component scores were tested using independent-samples comparisons, with Benjamini–Hochberg false discovery rate correction applied across muscle × component tests.

**Results:**

After FDR correction, significant between-group differences were identified in four muscle–component combinations. In the support leg, significant differences were observed in PC1 of the left tibialis anterior (p_FDR = 0.001, Cohen’s d = 1.726) and left rectus femoris (p_FDR = 0.029, Cohen’s d = 1.058), whereas the left lateral gastrocnemius differed in PC2 (p_FDR = 0.040, Cohen’s d = 0.943). In the swing leg, the right biceps femoris differed in PC1 (p_FDR = 0.015, Cohen’s d = 1.206).

**Conclusion:**

Elite and youth taekwondo athletes showed distinct muscle-specific and phase-dependent neuromuscular organization during the roundhouse kick, particularly in support-leg control and swing-leg posterior-chain regulation. FPCA revealed whole-waveform differences that were not captured by conventional discrete measures, providing useful evidence for technique analysis, youth athlete development, and more targeted taekwondo training.

## 1 Introduction

The taekwondo roundhouse kick is one of the most frequently used and performance-relevant attacking techniques in modern taekwondo. Its execution requires rapid swing-leg acceleration, coordinated hip–knee–ankle motion, and stable support-leg control within a short movement duration. Previous biomechanical studies have shown that effective roundhouse kicking is closely associated with rapid pelvic rotation, hip abduction and flexion, knee extension velocity, impact force, and execution time, indicating that the technique depends on both proximal rotational control and distal limb coordination (Falco et al., 2009; Gavagan and Sayers, 2017; Vagner et al., 2023). Because the movement involves a rapid transition from kick initiation to swing-leg acceleration, target contact, and recovery, the roundhouse kick provides a useful sport-specific model for examining neuromuscular activity across a complete kicking cycle.

From a neuromuscular perspective, the roundhouse kick places different functional demands on the support and swing limbs. The support limb must maintain single-leg stability, regulate foot–ankle posture, and provide the mechanical base for stance-phase rotation and lower-limb power generation (Jandačka et al., 2013), whereas the swing limb must coordinate knee lift, forward acceleration, and terminal control before impact (Jung and Park, 2022). These demands require coordinated activation of muscles involved in ankle stabilization, knee control, hip movement, and thigh–shank coordination. Previous studies have reported that lower-limb muscle activation during roundhouse kicking differs according to skill level, kicking height, and task condition, suggesting that muscle activation patterns are sensitive to both technical demand and athlete experience (Valdés-Badilla et al., 2018; Chang et al., 2021; Barramuño-Medina et al., 2025; Sun et al., 2024). However, most existing evidence has focused on selected muscles, isolated time points, or discrete EMG outcomes, which limits understanding of how activation patterns evolve across the whole kicking cycle.

Surface electromyography has been widely used to examine muscle activity during taekwondo kicking, but previous roundhouse-kick studies have commonly relied on discrete variables, such as peak amplitude, mean RMS, integrated EMG, reaction time, or the timing of maximal activation, to describe muscle activity during kicking tasks (De Luca, 1997; Valdés-Badilla et al., 2018; Chang et al., 2021; Ervilha et al., 2020; Sun et al., 2024). Although these variables are useful for summarizing selected features of muscle activation, they reduce the continuous sEMG signal to isolated values or predefined intervals and may therefore provide limited information about waveform-level changes across the kicking cycle. This limitation is important because muscle activation during the roundhouse kick is likely to vary across movement phases, including support establishment, swing-leg acceleration, target contact, and recovery. Waveform-based approaches have been recommended for biomechanical and EMG time-series data because they preserve the temporal structure of the signal and can identify differences that may be missed by discrete summary measures (McKay et al., 2013; Robinson et al., 2015; Warmenhoven et al., 2021). This issue is especially relevant when comparing elite and youth athletes, because differences in age, training experience, and neuromuscular development may influence not only the magnitude of activation but also the temporal organization and shape of muscle-activation waveforms.

Functional principal component analysis provides a waveform-based approach for identifying dominant modes of variation in continuous biomechanical and electromyographic signals. Instead of reducing EMG activity to a small number of discrete outcomes, FPCA characterizes how entire time-normalized waveforms vary across participants and groups. In biomechanical waveform analysis, leading components often capture dominant amplitude-related or cycle-wide variation, whereas secondary components may reflect timing- or shape-related redistribution across the movement cycle (Dona et al., 2009; Brandon et al., 2013; Warmenhoven et al., 2021). Therefore, applying FPCA to sEMG waveforms during the roundhouse kick may reveal muscle-specific and phase-dependent differences that are not apparent from conventional discrete EMG measures.

Accordingly, this study aimed to examine trunk and lower-limb sEMG waveform differences during the taekwondo roundhouse kick between elite and youth athletes using FPCA. Specifically, this study sought to identify whether between-group differences were expressed in dominant amplitude-related or cycle-wide waveform modes or in secondary timing-/shape-related modes across the kicking cycle. Based on the technical demands of the roundhouse kick and previous evidence on experience-related differences in kicking biomechanics and EMG activity, we expected that elite and youth athletes would show muscle-specific differences in both support-leg and swing-leg activation patterns.

## 2 Methods

### 2.1 Data collection

This study was approved by the Ethics Committee of Universiti Sains Malaysia (USM/JEPeM/25030312). This study was registered at ClinicalTrials.gov (Identifier: NCT07307378) prior to manuscript submission. All procedures were conducted in accordance with the approved ethical guidelines, and written informed consent was obtained from all participants (for youth athletes, consent was obtained from the participants and their legal guardians).

A sensitivity analysis was performed in G*Power 3.1 for an independent-samples t-test (two-tailed, α = 0.05, power = 0.80, allocation ratio = 1). A total of 38 male taekwondo athletes were recruited and allocated to an elite group (n = 19) and a youth group (n = 19). Elite athletes were included if they: (i) achieved a podium finish at national-level competitions or ranked within the top eight at international-level competitions; (ii) were aged 18–28 years; (iii) had at least 6 years of taekwondo training; and (iv) were free from injury and substance dependence. Youth athletes were included if they: (i) achieved a podium finish at provincial-level competitions or won a championship at city-level competitions; (ii) were aged 14–17 years; (iii) had at least 2 years of taekwondo training; and (iv) were free from injury and substance dependence.

### 2.2 Instrumentation and experimental procedure

**FIGURE 1:**
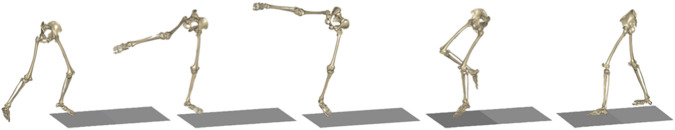
Schematic illustration of the roundhouse kick.

Based on previous research and the technical characteristics of the roundhouse kick, surface electromyography electrodes were placed bilaterally over seven trunk and lower-limb muscles in accordance with the SENIAM recommendations, including one trunk muscle, the rectus abdominis, and six lower-limb muscles: gluteus maximus, rectus femoris, biceps femoris, semitendinosus, lateral gastrocnemius, and tibialis anterior (Sun et al., 2024). In addition, kinematic data were recorded using a 12-camera Vicon three-dimensional motion capture system (Oxford Metrics Ltd., UK) at a sampling frequency of 200 Hz, while sEMG signals were acquired simultaneously using the electromyography system at 2,000 Hz. The two systems were hardware-synchronized via a shared analog interface, allowing both recordings to start with the same timing reference. Synchronization was verified during post processing using the common trigger marker before the data were segmented and time-normalized for analysis. The roundhouse-kick task and corresponding movement sequence are illustrated in Figure 1.

All participants were required to wear tight-fitting pants on the testing day. Before data collection, the testing procedures, movement requirements, and safety instructions were explained to all participants, and basic anthropometric measurements were recorded. A standardized 15-min warm-up was then performed, consisting of dynamic stretching and task-specific preparatory exercises to reduce the risk of injury.

Data collection was conducted during the preparatory phase of the athletes’ annual training macrocycle. During this period, athletes were engaged in regular technical, physical, and taekwondo-specific training, but were not within an immediate competition week. This timing was selected to reduce the potential influence of acute competition-related fatigue or tapering on sEMG waveform characteristics.

The experiment was conducted in the biomechanics laboratory of a local university in China. All participants were tested and instructed by the researcher together with trained laboratory staff. During preparation, reflective markers were placed according to the Vicon Plug-in Gait lower-body marker set. The marker set included bilateral anterior superior iliac spine markers (LASI and RASI), posterior superior iliac spine markers (LPSI and RPSI), thigh markers (LTHI and RTHI), knee markers (LKNE and RKNE), tibia markers (LTIB and RTIB), ankle markers (LANK and RANK), heel markers (LHEE and RHEE), and toe markers (LTOE and RTOE). The marker placement used for kinematic reconstruction is illustrated in Supplementary Figure S1. The Plug-in Gait lower-body model in Vicon Nexus was used to reconstruct the pelvis, thigh, shank, and foot segments. The pelvis segment was reconstructed from LASI, RASI, LPSI, and RPSI. The thigh segments were reconstructed using the thigh and knee markers, the shank segments using the knee, tibia, and ankle markers, and the foot segments using the ankle, heel, and toe markers, following the Plug-in Gait segment-definition conventions. The reconstructed kinematic data were used primarily for movement-cycle identification, trial segmentation, synchronization with the sEMG recordings, and time normalization of the corresponding sEMG waveforms. Kinematic variables were not treated as primary outcome variables in the present FPCA analysis. During testing, all participants were right-leg dominant and performed the roundhouse kick with the right leg as the kicking limb. Accordingly, the left limb was defined as the support leg and the right limb as the swing leg throughout the analysis. Participants performed the roundhouse kick in response to verbal instructions from the researcher and were asked to strike a designated target positioned at head height relative to each participant. Kicking performance was monitored throughout testing, and data collection continued until six valid kicking trials had been completed.

### 2.3 EMG preprocessing

Raw EMG signals were processed using Noraxon MR3 software (version 3.16). Signals were band-pass filtered (20–450 Hz), full-wave rectified, and smoothed using a root mean square (RMS) algorithm with a 50 ms moving window to obtain the linear envelope. Each trial was segmented according to the movement cycle and time-normalized to 0%–100%. The normalized EMG waveforms were interpolated to 101 equidistant points to ensure comparability across participantss. To further reduce high-frequency noise and improve signal continuity, a Savitzky–Golay smoothing filter was applied to the interpolated signals (De Luca et al., 2010). To allow between-participants comparison, signals were analyzed in their time-normalized form without additional amplitude normalization, consistent with FPCA-based waveform analysis. These preprocessing steps were performed to obtain a smooth and comparable representation of muscle activation patterns across the movement cycle.

The primary FPCA was conducted on time-normalized sEMG linear envelopes without additional amplitude normalization. This decision was made because the main objective of the study was to characterize the dominant modes of waveform variation across the roundhouse-kick cycle, including amplitude-related variation in the overall sEMG profile. Applying an additional amplitude-normalization procedure before FPCA could reduce or remove between-participant differences in overall signal magnitude, which may represent an important source of waveform variability captured by the leading principal components. Therefore, non-amplitude-normalized waveforms were retained for the primary analysis to preserve both magnitude-related and timing-/shape-related sources of variation across the kicking cycle.

### 2.4 Functional principal component analysis

FPCA was applied to the time-normalized EMG waveforms to characterize the dominant patterns of variation across the movement cycle (Coffey et al., 2011; Warmenhoven et al., 2018). FPCA represents each waveform *Xi*(*t*) via a Karhunen–Loève expansion, decomposing it into a mean function and a weighted sum of orthogonal eigenfunctions (functional principal components) that capture dominant modes of variability (Lee et al., 2019; Jiang et al., 2023; Warmenhoven et al., 2021). Each waveform was treated as a continuous function and represented as:

$$Xi\left(t\right)=\mu \left(t\right)+\sum_{k=1}^{K}\xi k\phi k\left(t\right)+\epsilon i\left(t\right)$$

where *μ*(*t*) denotes the mean function, *ϕk* (*t*) represents the *k*th eigenfunction, and *ξ*k is the corresponding score for each participant, and $\epsilon_{i}\left(t\right)$ denotes the residual term.

Prior to decomposition, all waveforms were mean-centered across participantss. FPCA was implemented using singular value decomposition (SVD), which is mathematically equivalent to principal component analysis applied to discretized functional data. The number of retained principal components was determined based on cumulative explained variance, with a threshold set at 95%. The principal components meeting this criterion were retained for further analysis (Warmenhoven et al., 2021; Wang et al., 2016).

The interpretation of principal components followed established conventions in functional data analysis (Deluzio et al., 1997). Components with eigenfunctions exhibiting a consistent sign across the time domain were interpreted as representing amplitude-related variations, whereas components with oscillatory patterns were considered to reflect temporal or shape-related variations in muscle activation (Ramsay, J et al., 2009).

### 2.5 Statistical analysis

For each muscle, FPCA scores of the retained principal components were extracted for all participants. The number of retained components was determined by the cumulative explained variance criterion (≥95%). Between-group differences were assessed on the first K component scores (up to a maximum of 6 components per muscle), as these components captured the dominant waveform variability. All hypothesis tests were two-tailed. For each muscle × component combination, group differences in FPCA scores were evaluated using an independent-samples t-test with unequal variances (Welch’s t-test), which is robust to heteroscedasticity and is appropriate for group comparisons when equal-variance assumptions may not hold. Normality of FPCA scores was inspected using distributional diagnostics and formal tests where appropriate. Results were summarized as mean ± SD of FPCA scores for each group, together with the corresponding test statistics (t) and unadjusted p-values.

To control for inflated Type I error arising from multiple testing across muscles and principal components, p-values from all muscle × component tests were adjusted using the Benjamini–Hochberg false discovery rate (BH-FDR) procedure with q = 0.05. Both unadjusted p-values (p) and FDR-adjusted p-values were reported. Statistical significance was defined as p-FDR < 0.05. Effect sizes were quantified using Cohen’s d for each tested component score, calculated as the difference in group means divided by the pooled standard deviation. Effect sizes were interpreted using conventional thresholds (small ≈ 0.2, moderate ≈ 0.5, large ≥ 0.8) to facilitate practical interpretation beyond statistical significance. All analyses were performed in MATLAB 2024b (MathWorks Inc., Natick, MA, USA) using a custom SVD-based FPCA implementation and in-house statistical scripts. The analysis code and parameter settings (e.g., smoothing method, variance-retention threshold, and FDR level) were fixed *a priori* and applied consistently across all muscles.

## 3 Results

### 3.1 Basic information of the participants

Based on competitive level and training background, the participants were categorized into an elite group and a youth group. Detailed demographic characteristics of the two groups are presented in Table 1. Specifically, the elite group had a mean age of 24.89 ± 1.85 years, height of 181.89 ± 3.05 cm, body weight of 69.26 ± 11.11 kg, BMI of 20.91 ± 1.43 kg/m^2^, and training experience of 11.10 ± 1.76 years. In comparison, the youth group had a mean age of 16.05 ± 0.85 years, height of 178.79 ± 3.07 cm, body weight of 64.32 ± 5.49 kg, BMI of 20.09 ± 1.28 kg/m^2^, and training experience of 2.37 ± 0.76 years. Descriptively, the elite athletes were older and more experienced than the youth athletes, and they also showed slightly higher mean values for height, body weight, and BMI.

**TABLE 1:** Demographic and anthropometric characteristics of the participants.

Group	Age	Height	Weight	BMI	Years of experience
Elite	24.89 ± 1.85	181.89 ± 3.05	69.26 ± 11.11	20.91 ± 1.43	11.1 ± 1.76
Youth	16.05 ± 0.85	178.79 ± 3.07	64.32 ± 5.49	20.09 ± 1.28	2.368 ± 0.76

### 3.2 Overall between-group differences in FPCA scores

Table 2 summarizes between-group comparisons of FPCA scores derived from time-normalized sEMG waveforms. Significant between-group differences in FPCA scores were observed for four muscle–component combinations after Benjamini–Hochberg false discovery rate (FDR) correction across all muscle–component comparisons. For the left rectus femoris, the first principal component (PC1) score differed significantly between groups (p_FDR = 0.029; Cohen’s d = 1.058), with a positive effect size indicating higher PC1 scores in the elite group. PC1 accounted for 94.6% of the total waveform variance, suggesting that the group difference was captured by the dominant mode of variability in this muscle’s sEMG profile. Similarly, the right biceps femoris exhibited a significant between-group difference in PC1 scores (p_FDR = 0.015; Cohen’s d = 1.206), again with higher scores in the elite group. PC1 explained 91.6% of the variance, indicating that the between-group separation was primarily driven by the leading pattern of waveform variability. For the left tibialis anterior, PC1 scores also differed significantly between groups (p_FDR = 0.001; Cohen’s d = 1.726), with a large positive effect size showing higher PC1 scores in the elite group. PC1 accounted for 94.9% of the variance, demonstrating that the group difference was concentrated in the principal source of waveform variation for this muscle. In contrast, the left lateral gastrocnemius showed a significant between-group difference in the second principal component (PC2) score (p_FDR = 0.040; Cohen’s d = 0.943). PC2 explained 5.0% of the total variance, suggesting that the between-group difference was expressed in a secondary, yet interpretable, mode of waveform variability. Based on the eigenfunction patterns and the component interpretation criteria described in the Methods section, the significant PC1 findings in the left rectus femoris, left tibialis anterior, and right biceps femoris were interpreted primarily as dominant amplitude-related or cycle-wide waveform differences. These components explained a large proportion of the total waveform variance and reflected broad differences in the overall sEMG profile across the kicking cycle. In contrast, the significant PC2 finding in the left lateral gastrocnemius was interpreted primarily as a timing-/shape-related waveform difference. This component represented a secondary mode of variation and reflected a phase-dependent redistribution of activation rather than a uniform amplitude shift across the whole cycle.

**TABLE 2:** Between-group comparisons of FPCA score(s) for each muscle and principal component (PC) derived from time-normalized sEMG waveforms.

Muscle	PC	Explained_%	t	*p*	*p*_FDR	Cohens_d
RA-L	1	99.09014032	2.012	0.059	0.206	0.671
RA-R	1	95.8222082	0.577	0.569	0.773	0.192
Gmax-L	1	99.77917241	1.224	0.237	0.497	0.408
Gmax-R	1	99.91207322	−0.561	0.582	0.773	−0.186
RF-L	1	94.61437118	3.175	0.004	0.029*	1.058
RF-L	2	4.127472258	−2.016	0.051	0.206	−0.672
RF-R	1	99.75755921	1.103	0.284	0.542	0.368
BF-L	1	90.06173202	−1.398	0.172	0.451	−0.466
BF-L	2	6.461077911	0.493	0.626	0.773	0.164
BF-R	1	91.63245319	3.619	0.001	0.015*	1.206
BF-R	2	4.729010723	−1.841	0.079	0.236	−0.614
ST-L	1	95.19472216	0.009	0.992	0.992	0.003
ST-R	1	96.06655216	0.008	0.992	0.993	0.002
GA-L	1	92.97555406	−0.539	0.593	0.773	−0.179
GA-L	2	5.027854988	2.830	0.007	0.040*	0.943
GA-R	1	94.98139431	−0.044	0.964	0.992	−0.014
GA-R	2	2.98360083	−1.238	0.223	0.497	−0.412
TA-L	1	94.92317278	5.179	0.001	0.001**	1.726
TA-L	2	3.529272844	−0.229	0.820	0.957	−0.076
TA-R	1	89.51810773	0.842	0.407	0.658	0.280
TA-R	2	7.257430515	−0.973	0.337	0.589	−0.324

Explained_% denotes the variance explained by each PC. t and p are from two-tailed Welch’s independent-samples t-tests on PC, scores (Elite vs. Youth). p_FDR, indicates Benjamini–Hochberg false discovery rate–adjusted p-values across all muscle × PC, tests, and statistically significant results are highlighted (p_FDR <0.05). Cohen’s d quantifies the standardized between-group difference in PC, scores (positive values indicate Elite > Youth; negative values indicate Elite < Youth). Muscle abbreviations: RA, rectus abdominis; GMax, gluteus maximus; RF, rectus femoris; BF, biceps femoris; ST, semitendinosus; GA, gastrocnemius (lateral head); TA, tibialis anterior. Side denotes left (L) and right (R).

* p_FDR < 0.05.

** p_FDR < 0.01.

A sensitivity analysis was performed using task-specific peak-normalized sEMG waveforms to evaluate whether the primary FPCA findings were robust to amplitude scaling. After peak normalization, the four significant muscle–component findings identified in the primary analysis remained significant after FDR correction. Specifically, significant between-group differences were retained for the left rectus femoris PC1 (explained variance = 91.54%, t = 4.307, p = 0.001, p_FDR = 0.020, Cohen’s d = 1.397), left tibialis anterior PC1 (explained variance = 88.47%, t = 3.825, p = 0.001, p_FDR = 0.007, Cohen’s d = 1.241), right biceps femoris PC1 (explained variance = 91.11%, t = 3.286, p = 0.002, p_FDR = 0.016, Cohen’s d = 1.066), and left lateral gastrocnemius PC2 (explained variance = 16.93%, t = 3.026, p = 0.005, p_FDR = 0.028, Cohen’s d = 0.981). These results indicate that the main FPCA-derived findings were not solely attributable to inter-individual differences in raw sEMG magnitude. Full results of the sensitivity analysis are provided in Supplementary Table S1.

### 3.3 Support-leg muscle activation patterns across the kicking cycle

**FIGURE 2:**
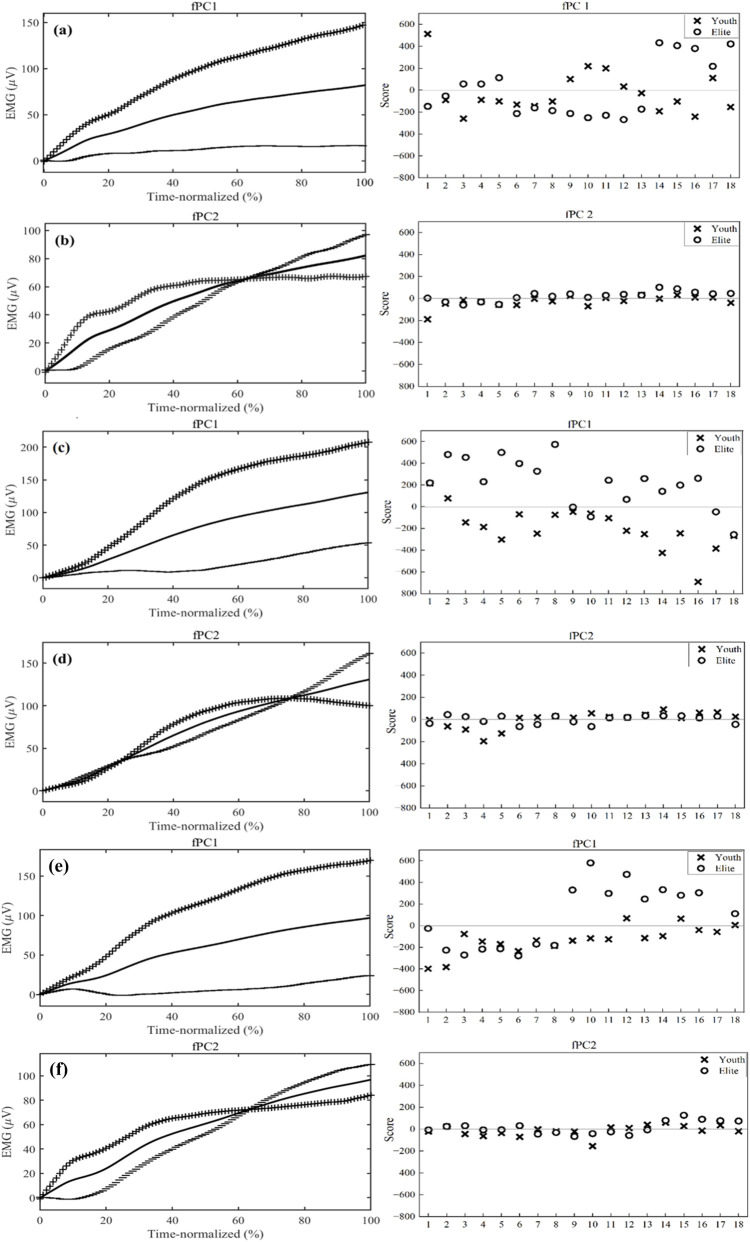
Functional principal component analysis of support-leg muscle activation patterns across the time-normalized kicking cycle. Note: **(a,b)** show the left lateral gastrocnemius, **(c,d)** show the left tibialis anterior, and **(e,f)** show the left rectus femoris. For each muscle, the left panel presents the reconstructed waveforms associated with PC1 and PC2 across the time-normalized kicking cycle (0%–100%), whereas the right panel shows the corresponding individual component scores for the youth and elite groups. Significant between-group differences were identified in PC2 for the left lateral gastrocnemius and in PC1 for both the left tibialis anterior and left rectus femoris after FDR correction.

Figure 2 presents the FPCA results for the support-leg muscles in elite and youth taekwondo athletes across the time-normalized kicking cycle. A clear sequential pattern of between-group differences was observed. In the earlier portion of the support phase, significant differences were primarily captured by the dominant component (PC1) of the left tibialis anterior and left rectus femoris. For the left tibialis anterior, PC1 accounted for the largest proportion of waveform variance, and its scores differed significantly between groups after FDR correction (*p*_*FDR* = 0.001, Cohen’s d = 1.726). The reconstructed waveform showed that the divergence between groups became apparent early in the kicking cycle and remained evident across much of the support phase, indicating a robust difference in the dominant amplitude-related or cycle-wide waveform pattern. A similar result was observed for the left rectus femoris, in which PC1 also explained the dominant proportion of waveform variance and differed significantly between groups (p_FDR = 0.029, Cohen’s *d* = 1.058). Visual inspection of the reconstructed waveform suggested that separation between groups emerged at approximately 10% of the kicking cycle and became more pronounced through the middle and later portions of the movement. In contrast, the left lateral gastrocnemius exhibited a different temporal pattern. Although PC1 explained the largest proportion of waveform variance, no significant between-group difference was detected for PC1 scores. Instead, a significant difference was identified in PC2 after FDR correction (p_FDR = 0.040, Cohen’s *d* = 0.943), indicating that the group difference in this muscle was captured by a secondary mode of variation rather than by the dominant waveform pattern. The reconstructed PC2 waveform further suggested that this difference was concentrated mainly in the mid-to-late portion of the kicking cycle (approximately 23%–76%), with the greatest separation occurring around 45%, reflecting a phase-specific redistribution of activation across the support phase rather than a simple overall amplitude difference.

### 3.4 Swing-leg muscle activation patterns across the kicking cycle

**FIGURE 3:**
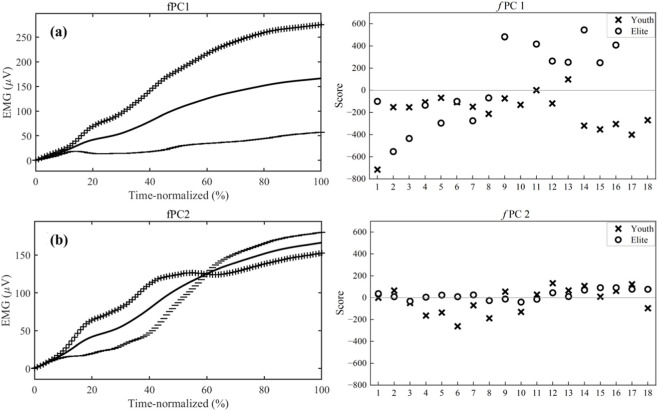
Functional principal component analysis of swing-leg biceps femoris activation across the time-normalized kicking cycle. Note: **(a,b)** show the right biceps femoris of the swing leg. For this muscle, the left panel presents the reconstructed waveforms associated with PC1 and PC2 across the time-normalized kicking cycle (0%–100%), whereas the right panel shows the corresponding individual component scores for the youth and elite groups. A significant between-group difference was identified in PC1 for the right biceps femoris after FDR correction, whereas no clear between-group separation was observed for PC2.

Figure 3 presents the FPCA results for the right biceps femoris of the swing leg in elite and youth taekwondo athletes across the time-normalized kicking cycle. The between-group difference was primarily captured by the dominant component (PC1). For the right biceps femoris, the first functional principal component accounted for the largest proportion of waveform variance, and its scores differed significantly between groups after FDR correction (*p*_FDR = 0.015, Cohen’s d = 1.206). Inspection of the reconstructed waveforms showed that the two groups were broadly comparable during the initial phase of the movement (0%–11%). From approximately 12% of the kicking cycle onward, waveform separation gradually emerged, reaching a first peak at around 18%, followed by a brief plateau, and then a second peak at approximately 40%. These two characteristic time points corresponded to the phase after toe-off of the swing leg, when the knee was being lifted and knee flexion approached its minimum angle, and to the hip-turning phase characterized by hip abduction, respectively. Overall, the between-group difference became apparent early in the kicking cycle, increased progressively as the movement advanced, and remained clearly evident through the middle and later portions of the swing phase. This pattern indicates that the between-group difference in the swing-leg biceps femoris was mainly reflected in the dominant amplitude-related or cycle-wide waveform pattern across the kicking cycle rather than in a secondary waveform feature. In contrast, PC2 explained a smaller proportion of waveform variation, and its score distributions showed substantial overlap between groups, indicating that this component contributed little to group discrimination.

## 4 Discussion

### 4.1 Principal findings and contribution to current understanding

The present study examined lower-limb sEMG waveform differences during the taekwondo roundhouse kick between elite and youth athletes using FPCA. The main findings were that significant between-group differences were identified in four muscle–component combinations after FDR correction: PC1 of the support-leg left tibialis anterior, PC1 of the support-leg left rectus femoris, PC2 of the support-leg left lateral gastrocnemius, and PC1 of the swing-leg right biceps femoris. These findings indicate that group differences were not limited to the kicking limb, but were distributed across both support- and swing-limb muscles. This pattern supports the view that the roundhouse kick should be understood as a whole-body and bilateral lower-limb skill (Xu et al., 2025), in which support-leg regulation and swing-leg acceleration are both relevant to movement organization.

Previous biomechanical studies have established that the taekwondo roundhouse kick is influenced by execution time, impact force, pelvic rotation, lower-limb kinematics, and segmental contributions to toe velocity (Falco et al., 2009; Gavagan and Sayers, 2017; Jung and Park, 2022; Vagner et al., 2023). EMG-based studies have further shown that muscle activation during roundhouse kicking differs according to skill level, kicking height, and task condition (Valdés-Badilla et al., 2018; Chang et al., 2021; Barramuño-Medina et al., 2025; Sun et al., 2024). However, much of the existing evidence has relied on discrete EMG variables, selected muscles, or isolated time points. Although such approaches are useful for describing peak amplitude, mean RMS, integrated EMG, or activation timing, they provide limited information about how muscle activation varies across the complete kicking cycle. The present study extends this body of work by applying FPCA to time-normalized sEMG waveforms, thereby distinguishing dominant cycle-wide waveform differences from secondary timing-shape-related waveform differences.

The component-specific findings are important for interpretation. The significant PC1 findings in the left tibialis anterior, left rectus femoris, and right biceps femoris were interpreted as dominant amplitude-related or cycle-wide waveform differences because these components explained the largest proportion of waveform variance. In contrast, the left lateral gastrocnemius showed a significant PC2 difference, suggesting a secondary timing-shape-related redistribution of activation rather than a broad cycle-wide difference. The task-specific peak-normalized sensitivity analysis further showed that the four significant findings remained significant after amplitude normalization, indicating that the main results were not solely attributable to raw sEMG magnitude. Nevertheless, these findings should be interpreted cautiously as FPCA-derived waveform differences, not as direct evidence of greater neural drive, superior neuromuscular control, or specific mechanical advantage. This caution is consistent with previous work emphasizing that surface EMG amplitude is affected by multiple physiological and non-physiological factors and should not be used alone to infer neural strategies or muscle coordination mechanisms (Hug, 2011; Farina et al., 2014; Martinez-Valdes et al., 2018).

The developmental and training context of the two groups should also be considered. The elite athletes were older and had substantially longer taekwondo experience than the youth athletes, whereas the youth athletes were still within a period of biological growth and neuromuscular development. Therefore, the observed differences are unlikely to reflect competitive level alone. Rather, they may reflect the combined influence of maturation, muscle development, accumulated practice, coordination capacity, and task-specific experience. For this reason, the present findings are best interpreted as age- and level-related neuromuscular waveform characteristics during a sport-specific kicking task.

### 4.2 Support-leg neuromuscular organization

The support-leg findings suggest that support-side neuromuscular organization is a key feature distinguishing elite and youth athletes during the roundhouse kick. Three of the four significant findings were observed in the support leg, involving the tibialis anterior, rectus femoris, and lateral gastrocnemius. This result is meaningful because the support limb is responsible for maintaining single-leg stability, regulating foot–ankle posture, and providing a mechanical base for body rotation while the swing limb accelerates toward the target. Previous studies have emphasized the role of pivot-leg mechanics, support-leg control, and lower-limb coordination in roundhouse kicking (Jandačka et al., 2013; Chang et al., 2021; Vagner et al., 2023). The present FPCA findings add waveform-level evidence that support-leg muscle activation is not merely a background stabilizing factor, but an important part of the neuromuscular organization of the kick.

The left tibialis anterior showed a significant PC1 difference, indicating a dominant cycle-wide difference in the support-leg dorsiflexor waveform profile. Because the tibialis anterior contributes to dorsiflexion, foot positioning, and ankle control, this finding may reflect differences in how elite and youth athletes regulate the support foot during the kick. In the roundhouse kick, the support foot must remain responsive while the body rotates and the swing limb accelerates. A distinct tibialis anterior waveform profile may therefore be related to differences in support-foot preparation, ankle positioning, and dynamic postural regulation across the movement cycle. This interpretation is compatible with broader evidence that ankle-related muscles contribute to foot posture regulation and dynamic stability during movement tasks (De Ridder et al., 2015; Medina McKeon and Hoch, 2019; Lin et al., 2021; Sanchez-Morilla et al., 2025). Importantly, the present result does not indicate a single isolated time-point difference; rather, it suggests a broad difference in the dominant activation profile of the support-foot dorsiflexor.

The left rectus femoris also showed a significant PC1 difference, suggesting that the support-leg quadriceps-related waveform profile differed between groups across a large portion of the kicking cycle. As a biarticular muscle involved in hip flexion and knee extension, the rectus femoris may contribute to support-limb organization during kick initiation, body rotation, and movement recovery. Previous taekwondo studies have shown that lower-limb EMG activity differs by skill level and that thigh muscle activation is relevant to roundhouse-kick performance characteristics (Thibordee and Prasartwuth, 2014; Valdés-Badilla et al., 2018). In the present study, the rectus femoris finding suggests that support-leg knee- and hip-related activation should be considered when examining roundhouse-kick neuromuscular organization. However, because joint kinetics and direct mechanical output were not examined as primary outcomes, this result should be interpreted as a waveform-level group difference rather than direct evidence of a mechanical advantage.

The left lateral gastrocnemius showed a different pattern, with a significant group difference in PC2 rather than PC1. This finding is particularly informative because PC2 reflected a secondary timing-shape-related mode of waveform variation. The result suggests that the two groups differed in how gastrocnemius activation was redistributed across specific portions of the kicking cycle, rather than in a simple overall amplitude shift. Given the role of the lateral gastrocnemius in plantarflexion and ankle-foot regulation, this may reflect different strategies for organizing support-foot activity during the transition from kick initiation to the middle portion of the movement. This interpretation is consistent with previous work showing that pivot-leg mechanics and support-leg muscle activation vary with roundhouse-kick height and task demands (Chang et al., 2021). It also supports the broader view that effective taekwondo kicking depends on coordinated lower-limb actions rather than isolated swing-leg movement (Jia et al., 2024).

### 4.3 Swing-leg posterior-thigh activation

In the swing leg, the right biceps femoris showed a significant PC1 difference, indicating a dominant cycle-wide difference in the posterior-thigh waveform profile. The biceps femoris contributes to hip extension, knee flexion, and thigh–shank coordination (Bramah et al., 2024), all of which are relevant during swing-leg lift, forward acceleration, and terminal control before target contact. Previous biomechanical work has emphasized that toe velocity during the taekwondo roundhouse kick is influenced by coordinated contributions from multiple body segments (Jung and Park, 2022). Broader lower-limb biomechanics research also indicates that the biceps femoris contributes to knee motion regulation during dynamic movement (Arnold et al., 2007). The present finding therefore suggests that elite and youth athletes differed in the overall organization of posterior-thigh activation during the kicking cycle.

The reconstructed waveform pattern showed that the between-group separation became apparent after the initial portion of the movement and remained evident through the middle and later portions of the kicking cycle. This timing is functionally relevant because the swing limb must transition rapidly from knee lift to forward acceleration and preparation for contact. The biceps femoris may contribute to controlling the thigh and shank during this transition, especially as the limb moves from chambering toward the striking phase. However, because the present study did not directly measure joint moments, impact force, or segmental power, the biceps femoris result should not be interpreted as direct evidence that one group produced more effective kicking mechanics. Instead, it indicates that the posterior-thigh sEMG waveform profile differed between groups and may represent one neuromuscular feature associated with age- and level-related kicking organization.

Taken together with the support-leg findings, the swing-leg result indicates that group differences during the roundhouse kick were distributed across muscles with different functional roles. The support-leg tibialis anterior, rectus femoris, and lateral gastrocnemius were related to ankle-foot and knee-related support regulation, whereas the swing-leg biceps femoris was related to posterior-thigh organization during limb acceleration and control. This distribution suggests that FPCA can help identify how different muscles contribute to the temporal organization of a complex sport-specific movement.

Overall, the findings suggest that youth roundhouse-kick training should not focus only on swing-leg speed or final target contact. The observed waveform differences in support-leg ankle and quadriceps muscles indicate that support-side activation may deserve greater attention during technical development. Similarly, the swing-leg biceps femoris result suggests that posterior-thigh activation during the swing phase may be relevant to how the kicking limb is organized. For youth athletes, training approaches that progressively develop balance, single-leg support, ankle–knee control, and coordinated swing-leg activation may be useful, provided that they are adapted to the athlete’s developmental stage. This interpretation aligns with youth athlete development and neuromuscular training literature, which emphasizes progressive, developmentally appropriate training rather than direct imitation of adult or elite movement outputs (Bergeron et al., 2015; Myer et al., 2011; Varghese et al., 2022; Lutz et al., 2024).

### 4.4 Limitations and future directions

Several limitations should be considered when interpreting the present findings. First, the comparison between elite and youth athletes involved differences not only in competitive level and training background, but also in age, biological maturation, muscle development, coordination capacity, and accumulated practice experience. These factors are particularly relevant in youth sport research because neuromuscular function and movement coordination continue to develop during adolescence (Bergeron et al., 2015; Ford et al., 2011; Varghese et al., 2022). Therefore, the observed sEMG waveform differences should not be interpreted as a pure expertise effect. Instead, they are more appropriately understood as age- and level-related waveform characteristics observed during a sport-specific kicking task.

Second, the cross-sectional design prevents causal interpretation. The present results cannot determine whether the identified waveform differences resulted from long-term taekwondo training, biological maturation, individual motor development, or the interaction of these factors. Longitudinal studies following youth athletes across different stages of technical development would be needed to clarify whether FPCA-derived waveform features change systematically with training exposure and competitive progression.

Third, the interpretation of FPCA components remains indirect. Although FPCA is useful for identifying dominant and secondary modes of waveform variation, component interpretation depends on the shape of the eigenfunctions and reconstructed waveforms rather than on direct physiological measurement (Brandon et al., 2013; Warmenhoven et al., 2021). In the present study, PC1 findings were interpreted as dominant amplitude-related or cycle-wide waveform differences, whereas the gastrocnemius PC2 finding was interpreted as a timing-shape-related redistribution. However, these interpretations should not be taken as direct evidence of neural drive, motor-unit behaviour, or specific mechanical mechanisms. This caution is consistent with previous work showing that surface EMG amplitude is influenced by multiple physiological and non-physiological factors and cannot be used alone to infer neural drive or muscle coordination mechanisms (Hug, 2011; Farina et al., 2014; Martinez-Valdes et al., 2018).

Finally, the kicking task was performed under standardized laboratory conditions. This design improved experimental control but may not fully represent the tactical and environmental demands of competition, where opponent interaction, fatigue, repeated kicking, decision-making, and variable target distance can influence neuromuscular organization. Future research should examine FPCA-derived sEMG patterns under more ecologically valid conditions, including repeated-kick protocols, fatigue tasks, and opponent-based kicking situations.

## 5 Conclusion

This study showed that elite and youth taekwondo athletes differed in selected FPCA-derived sEMG waveform features during the roundhouse kick. Significant between-group differences were identified in the support-leg left rectus femoris, left tibialis anterior, and left lateral gastrocnemius, as well as in the swing-leg right biceps femoris. The PC1 findings in the rectus femoris, tibialis anterior, and biceps femoris indicate dominant amplitude-related or cycle-wide waveform differences, whereas the PC2 finding in the lateral gastrocnemius indicates a secondary timing-/shape-related waveform difference. The task-specific peak-normalized sensitivity analysis showed that these findings remained significant after amplitude normalization, suggesting that the main results were not solely driven by raw sEMG magnitude. However, these findings should be interpreted as age- and level-related sEMG waveform differences rather than as direct evidence of superior neuromuscular control, greater neural drive, or specific mechanical mechanisms. Future studies incorporating maturity assessment, anthropometric matching, kinetic outcomes, and direct performance measures are needed to clarify the functional significance of these waveform differences.

## Data Availability

The datasets presented in this study can be found in online repositories. The names of the repository/repositories and accession number(s) can be found in the article/Supplementary Material.
