# Supplementary material for: Lower-limb muscle activation patterns during the taekwondo roundhouse kick in elite and youth athletes: a functional principal component analysis
Source: Front Bioeng Biotechnol. 2026 Jun 11;14:1844590. doi: 10.3389/fbioe.2026.1844590 (PMC13294767; doi:10.3389/fbioe.2026.1844590)
Supplement: Supplementary file 1 [file Table1.docx]

Supplementary Table S1. Sensitivity analysis using task-specific peak-normalized sEMG waveforms.

| Muscle | PC | Explained (%) | t | *p* | *p*_FDR | Cohen’s d |
| --- | --- | --- | --- | --- | --- | --- |
| RF-L | PC1 | 91.54 | 4.307 | 0.001 | 0.002 | 1.397 |
| TA-L | PC1 | 88.47 | 3.825 | 0.001 | 0.007 | 1.241 |
| BF-R | PC1 | 91.11 | 3.2863 | 0.002 | 0.016 | 1.066 |
| GA-L | PC2 | 16.93 | 3.026 | 0.005 | 0.028 | 0.981 |

*Note: The sensitivity analysis was conducted using task-specific peak-normalized sEMG waveforms. For each participant, muscle, and side, the waveform was normalized to the maximum value observed for the corresponding muscle and side before FPCA was repeated. p_FDR values were obtained using Benjamini–Hochberg false discovery rate correction across the tested muscle × component comparisons. PC1 findings were interpreted as dominant amplitude-related or cycle-wide waveform differences, whereas the PC2 finding was interpreted as a secondary timing-/shape-related waveform difference.*
