# Supplementary figures and images for: Lower-limb muscle activation patterns during the taekwondo roundhouse kick in elite and youth athletes: a functional principal component analysis
Source: Front Bioeng Biotechnol. 2026 Jun 11;14:1844590. doi: 10.3389/fbioe.2026.1844590 (PMC13294767; doi:10.3389/fbioe.2026.1844590)

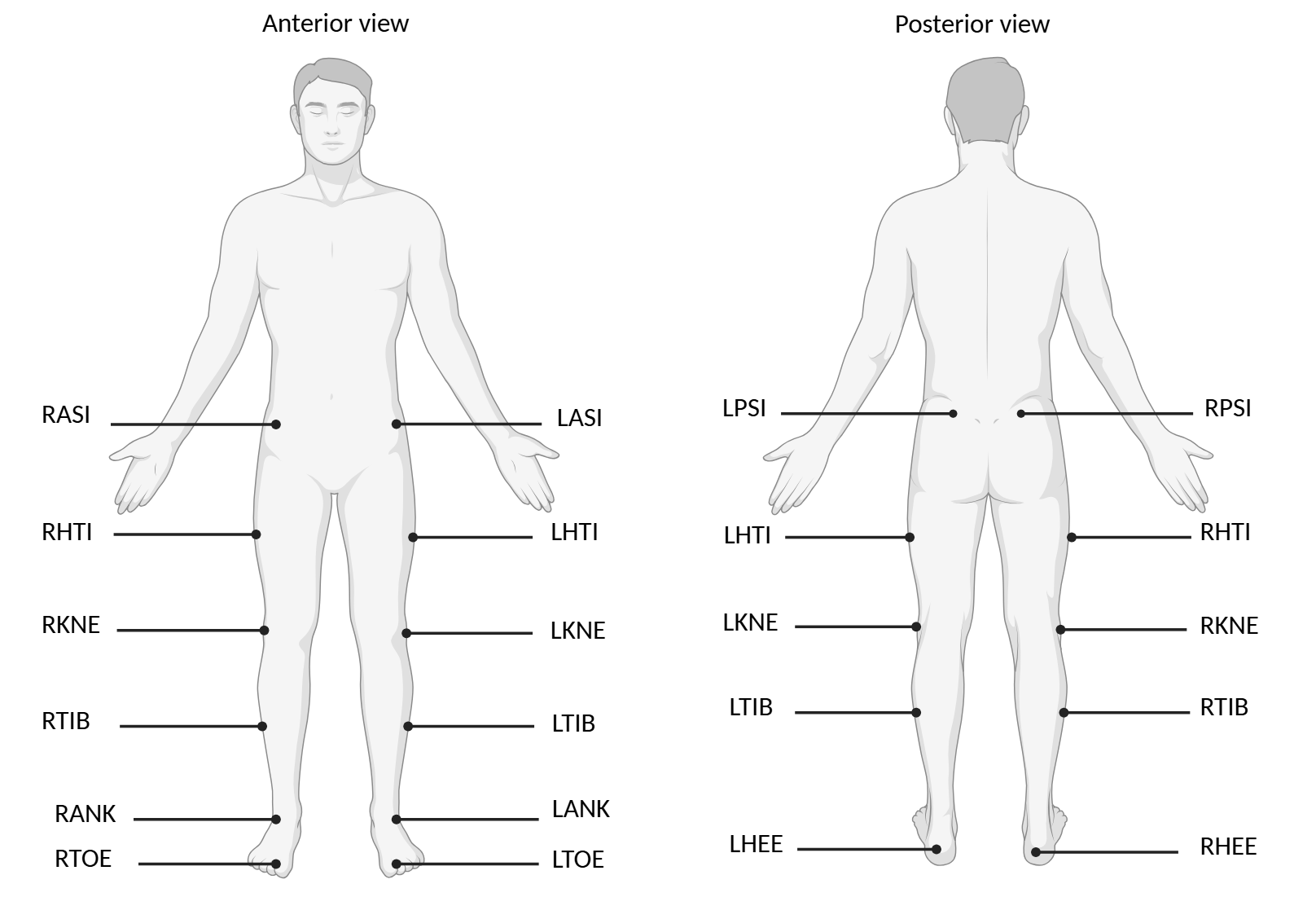

Supplement: Supplementary file 2 [file Image1.tif]
